# Polymorphisms in prostaglandin synthase 2/cyclooxygenase 2 (*PTGS2/COX2*) and risk of colorectal cancer

**DOI:** 10.1038/sj.bjc.6601906

**Published:** 2004-06-01

**Authors:** D G Cox, C Pontes, E Guino, M Navarro, A Osorio, F Canzian, V Moreno

**Affiliations:** 1 Genome Analysis Group, International Agency for Research on Cancer, 150 cours Albert Thomas, 69008 Lyon, Cedex 08, France; 2 Cancer Epidemiology Service, Catalan Institute of Oncology, Barcelona, Spain; 3 Medical Oncology Service, Catalan Institute of Oncology, Barcelona, Spain; 4 Digestive Surgery Service, Ciudad Sanitaria i Universitaria de Bellvitge, University of Barcelona, Barcelona, Spain

**Keywords:** *PTGS2/COX2*, polymorphisms, colorectal cancer, gene–environment interaction

## Abstract

Inflammation plays a key role in the development of colorectal cancers. We have investigated the relationship between *PTGS2* (*COX2*) polymorphisms and colorectal cancer risk in a hospital based case–control study. We recruited 292 patients with colorectal cancer and 274 controls from new patients admitted to Bellvitge Hospital, Barcelona, Spain, from 1996 to 1998. Subjects responded to a questionnaire on risk factors. Genotypes of the eight more frequent polymorphisms of *PTGS2* were determined. Two polymorphisms are located in the promoter sequence, one in the untranslated region of exon 1, one in exon 3, one in intron 5, two in the untranslated region of exon 10, and one downstream of the last polyadenylation (poly-A) signal. Associations were analysed with logistic regression models assuming a dominant effect for rare variants to increase statistical power. An association was detected between colorectal cancer and a polymorphism in the untranslated region of exon 10 of *PTGS2*, with an odds ratio (OR) of 2.49, 95% confidence interval (CI) of 1.17–5.32, *P*=0.01. A nearby polymorphism downstream of the last poly-A signal also showed a nonsignificant increase in risk (OR 2.17, 95% CI 0.99–4.78, *P*=0.05). Analysis of haplotypes confirmed that individuals with these variants were at increased risk of colorectal cancer (OR compared to the most frequent haplotype: 2.17, 95% CI 0.97–4.84, *P*=0.06) Interactions between *PTGS2* genotype and use of nonsteroidal anti-inflammatory drugs and risk of colorectal cancer were also explored.

Inflammation is becoming a major area of interest in the development and progression of many common cancers. Of particular interest is the involvement of inflammation in colorectal cancer. It is well established that inflammatory diseases of the colon, such as Crohn's disease and ulcerative colitis, substantially increase the risk of colorectal cancer (Lennard-Jones *et al*, 1977; Lashner *et al*, 1989; Rhodes and Campbell, 2002). Prostaglandins are molecules of particular interest in the inflammatory response. The cyclooxygenase (COX) enzymes, COX1 and COX2 (also known as prostaglandin synthases (PTGSs), PTGS1 and PTGS2), are the rate-limiting enzymes in the production of prostaglandins. PTGS2, which is induced in the inflammatory response, is responsible for the majority of prostaglandins present during the immune response to inflammation. Nonsteroidal anti-inflammatory drugs (NSAIDs) have been shown to protect against colorectal cancer (Gupta and Dubois, 2001). While the mechanism of this protection is still unclear, it can be hypothesised that it comes at least partly from limiting either the activity of PTGS2, or the amount of the enzyme present in the cell. It can also be hypothesised that genetic polymorphisms in *PTGS2* that result in alteration of the expression and/or the activity of the protein may modulate the inflammatory response, thus modifying the risk of colorectal cancer. While many single-nucleotide polymorphisms (SNPs) have been identified in the *PTGS2* gene (Halushka *et al*, 1999; Cox *et al*, 2001), few have been studied in terms of altering the function or expression of the enzyme (Fritsche *et al*, 2001; Lin *et al*, 2002). Only one polymorphism has been shown to alter the expression of *PTGS2* (Papafili *et al*, 2002). In this study, we have explored the impact of polymorphisms in *PTGS2* on risk of colorectal cancer as well as possible interactions between these polymorphisms and the use of NSAIDs.

## SUBJECTS AND METHODS

### Study population

A case–control study was conducted to assess risk factors of colorectal cancer. Cases were patients with a histologically confirmed new diagnosis of colorectal cancer attending the Bellvitge University Hospital in Barcelona, Spain, between January of 1996 and December 1998. This study includes 292 cases that could be interviewed and provide biological samples of adequate quality for genetic analyses.

Controls were randomly selected among patients admitted to the same hospital during the same period. Sex and broad age groups were used as stratifying criteria for frequency matching. From the daily patient admission lists, candidate controls were approached and, if they met these criteria, they were invited to participate. A total of 274 controls were analysed in this study. To avoid selection bias, the criterion for inclusion of controls was that the reason for the current admittance to the hospital should be a new disease (not previously diagnosed) for that patient. This criterion was used to avoid inclusion of patients with chronic diseases, who might be repeatedly admitted to hospital and modify their habits because of their disease. This procedure paralleled the criterion for cases who were also newly diagnosed incident cases. Both cases and controls had to have good mental condition, and be able to see and hear and follow an interview.

From a genetic point of view, we consider the hospitalised controls as being representative of the general Spanish population, as they came from very different hospital departments and included very different diseases. Moreover, it was recently reported in a pooled analysis with more than 15 000 controls from 73 different studies that the frequencies of genetic polymorphisms did not differ between hospitalised and population controls (Garte *et al*, 2001). No restriction criterion was imposed regarding the diagnosis of controls except those previously mentioned. The distribution of controls by diagnostic group was as follows: internal medicine 22%, acute surgery 19%, urology 17%, orthopedic surgery 15%, gastroenterology 16%, circulatory or respiratory 11%. In all, 43 controls (16%) had a diagnosis of inflammatory conditions that might be related to the studied polymorphisms: inflammatory bowel disease (*N*=2), peptic ulcer (*N*=2), pancreatitis (*N*=17), cholecystitis (*N*=16), arthritis (*N*=2) and diverticulitis (*N*=4). We compared the distribution of genotypes in this group and found no difference from the rest of the controls. Also, an analysis of the data excluding these controls yielded essentially the same results. Thus, we decided not to exclude any control that had been selected.

Cases and controls were personally interviewed by trained personnel using a structured questionnaire to determine demographic characteristics and potential risk factors for colorectal cancer. Long-term drug use was included and the focus in this analysis was on NSAIDs. Ever users were required to have consumed the drug regularly during at least 6 consecutive months. We analysed separately aspirin and other NSAIDs since in our population aspirin is often taken irregularly or at very low doses for secondary prevention of cardiovascular diseases, a fact that may mask some effects. Life-long history of alcohol consumption was collected through a structured questionnaire that requested type of beverage, average daily amount and duration. Average daily alcohol intake was estimated converting standard drink units to grams of pure alcohol. Also, a dietary questionnaire was administered that allowed estimation of total caloric intake.

All subjects were informed and gave written consent to participate in the study and to allow their biological samples to be genetically analysed, according to the Helsinki declaration. The study protocol was cleared by the Ethical Committee of the hospital.

### SNP genotyping

We selected *PTGS2* polymorphisms that were experimentally confirmed to exist (in our laboratory or in the literature) and that have sufficiently high frequency in Caucasians to allow a meaningful analysis. Eight SNPs were thus chosen for the study. Polymorphisms 401 and 926 (nomenclature of SNPs refers to base numbers in GenBank entry D28235; NCBI dbSNP accession numbers are reported in Table 2) are located in the promoter sequence, 1629 in the untranslated region of exon 1, 3050 in exon 3, 5209 in intron 5, 8473 and 9850 in the untranslated region of exon 10, and 10335 downstream of the last polyadenylation (poly-A) signal.

Cases and controls were randomised on PCR plates, so that an equal number of cases and controls could be analysed simultaneously. Polymorphisms 401, 3050, 5209, 8473 and 10335 were genotyped with the Invader assay (Third Wave Technologies, Madison, WI, USA). Polymorphisms 926 and 1629 were genotyped with TaqMan technology (ABI, Foster City, CA, USA). Polymorphism 9850 was genotyped by restriction enzyme digestion and electrophoresis on agarose gels. All samples that did not give a reliable result in the first round of genotyping were resubmitted to up to three additional rounds of genotyping. Data points that were still not filled after this procedure were left blank.

Sequences of primers and probes and experimental conditions are available online at http://www-gan.iarc.fr/PTGS2_G enotyping.html.

### Data analysis

For genotype data obtained with Invader or TaqMan technology, raw intensity counts were corrected with a negative control, and the ratio between the two signals (one colour for each allele) was used to call each genotype. Each polymorphism was tested in controls to ensure that it fitted the Hardy–Weinberg equilibrium. This was carried out using the Genotype Transposer (Cox and Canzian, 2001) and Arlequin (Schneider *et al*, 2000) software. *PTGS2* haplotypes were reconstructed by use of the PHASE version 2 software (Stephens and Donnelly, 2003).

To test the hypothesis of association between risk factors and genetic polymorphisms, multivariate methods based on logistic regression analyses were used. When cases were subdivided into groups by tumour site, polytomous logistic regression was used, comparing each group of cases to the whole set of controls. All analyses have been adjusted for age and sex. Odds ratios (OR) and 95% confidence intervals (CI) were calculated for each group. The *P*-values have been derived from likelihood ratio tests. Special attention was paid to explore the interactions between exposures and genotype. These were tested adding a product term of both variables (exposure and genotype) to the model that had the main effects. To increase statistical power for low-frequency variants, rare homozygotes were combined with heterozygotes assuming a dominant effect since their risk estimates were similar but less precise than the combination.

## RESULTS

Characteristics of the study population and the association with colorectal cancer are presented in Table 1

**Table 1:** Characteristics of cases and controls

	**Cases/controls**	**OR^a^**	**95% CI**	***P***-**value^b^**
*Sex*				
Male	173/140	1		
Female	119/134	0.73	(0.52–1.02)	
				
*Age (years)*				
(24, 62)	86/100	1		
(62, 73)	106/79	1.08	(0.53–2.17)	
(73, 92)	100/95	1.07	(0.38–2.99)	
				
*Family history*				
No relatives with cancer	137/152	1		0.001
Colon cancer	44/16	3.10	(1.65–5.81)	
Other cancers	111/106	1.26	(0.88–1.81)	
				
*Body mass index (BMI)*				
(16.0, 24.5)	107/78	1		0.21
(24.5, 27.6)	85/100	0.59	(0.39–0.90)	
(27.6, 40.7)	93/92	0.72	(0.47–1.11)	
				
*Smoking status*				
Non-smoker	151/157	1		0.61
Ex-smoker	91/65	1.11	(0.65–1.88)	
Smoker	50/52	0.85	(0.48–1.49)	
				
*Smoking duration (years)*				
Never	151/157	1		0.42
1–40	62/67	0.81	(0.47–1.38)	
>40	79/50	1.24	(0.71–2.17)	
				
*Alcohol daily intake (grams pure alcohol)*				
Never	100/123	1		0.015
1–60	141/125	1.46	(0.94–2.25)	
>60	51/26	2.69	(1.41–5.16)	
				
*Alcohol duration (years)*				
Never	100/123	1		0.014
1–40	70/77	1.19	(0.73–1.94)	
>40	122/74	2.00	(1.23–3.27)	
				
*Daily energy intake (kcal)*				
(530, 1636)	81/104	1		0.017
(1636, 2266)	99/86	1.45	(0.94–2.23)	
(2266, 5295)	105/80	1.68	(1.05–2.70)	
				
*Any NSAID*^c^				
Never	238/198	1		0.004
Ever	54/76	0.55	(0.37–0.83)	
				
*Aspirin*^c^				
Never	249/227	1		0.32
Ever	43/47	0.79	(0.50–1.25)	
				
*NSAID no aspirin*^c^				
Never	276/232	1		0.0001
Ever	16/42	0.31	(0.17–0.58)	
				
*Parity in women*				
Nulliparous	20/7	1		0.014
1	19/14	0.51	(0.17–1.56)	
2–3	51/79	0.24	(0.09–0.61)	
⩾4	29/34	0.34	(0.12–0.93)	

a Odds ratios adjusted for age and sex.

b *P*-values for trend except family history and smoking status.

c Ever users were required to have consumed the drug regularly during at least 6 consecutive months.

NSAIDs=nonsteroidal anti-inflammatory drugs; 95% CI=95% confidence interval.

. In this study, we find high risk of colorectal cancer associated to family history of colorectal cancer and to a lower extent of other cancers. Other risk factors are total energy intake and alcohol intake. Protective factors are high parity for women and long-term NSAID use. Use of NSAIDs in general was associated with significant reduction in risk. However, the reduction was stronger for NSAIDs other than aspirin. Use of aspirin alone was associated with a reduction in risk, but this observation was not statistically significant (Table 1).

All polymorphisms were in Hardy–Weinberg equilibrium. Tests for linkage disequilibrium (LD) between the polymorphisms showed that extensive LD exists along the entire gene (data not shown). Allele frequencies for all polymorphisms studied ranged from 1% for *PTGS2*.1629 to 31% for *PTGS2*.8473. We found that, in this sample set, carriers of the G allele of *PTGS2*.9850 had a significantly increased risk of colorectal cancer, with an OR, adjusted for age and sex, of 2.49 (95% CI 1.17–5.32; *P*-value=0.015). The minor allele of the SNP nearby, *PTGS2*.10335, was also associated with increased risk, with an OR of 2.17, approaching statistical significance (95% CI 0.99–4.78, *P*=0.05). Other polymorphisms studied were not related to colorectal cancer. The frequencies for each genotype or combination of genotypes and the corresponding odds ratios can be seen in Table 2

**Table 2:** Genotype counts for *PGTS2* polymorphisms and odds ratios (ORs) for colorectal cancer

	**Cases/controls^a^**	**OR^b^**	**95% CI**	***P*-value**
*PTGS2.401* (rs689465)^c^				
A/A	201/181	1		0.83
A/G	67/69	0.92	(0.62–1.38)	
G/G	9/9	0.78	(0.3–2.05)	
				
*PTGS2.926* (rs20417)				
G/G	150/170	1		0.87
G/C	59/77	0.92	(0.61–1.39)	
C/C	11/10	1.13	(0.46–2.80)	
				
*PTGS2.1629* (rs20424)				
C/C	280/263	1		0.38
C/G–G/G	10/6	1.59	(0.56–4.52)	
				
*PTGS2.3050* (rs5277)				
G/G	180/183	1		0.29
G/C	97/79	1.3	(0.9–1.87)	
C/C	13/10	1.5	(0.63–3.57)	
				
*PTGS2.5209* (rs20432)				
T/T	187/174	1		0.96
T/G	91/86	1.05	(0.73–1.52)	
G/G	12/11	0.99	(0.42–2.33)	
				
*PTGS2.8473* (rs5275)				
T/T	140/126	1		0.99
T/C	121/120	1.01	(0.71–1.45)	
C/C	29/25	1.05	(0.58–1.91)	
				
*PTGS2.9850* (rs4648298)				
A/A	257/258	1		0.01
A/G–G/G	24/10	**2.49**	**(1.17–5.32)**	
				
*PTGS2.10335* (rs689469)				
G/G	245/241	1		0.05
G/A–A/A	20/10	2.17	(0.99–4.78)	

a For some polymorphisms, numbers do not sum up to the totals of controls or cases due to genotyping failure. All samples that did not give a reliable result in the first round of genotyping were resubmitted to up to three additional rounds of genotyping. Data points that were still not filled after this procedure were left blank.

b Odds ratio adjusted for age and sex. For the low-frequency variants, rare homozygotes have been combined with heterozygotes since their risk estimates were similar but less precise than the combination.

c Nomenclature of single-nucleotide polymorphisms (SNPs) refers to base numbers in GenBank entry D28235. NCBI dbSNP (http://www.ncbi.nlm.nih.gov/SN P/) accession numbers are reported in parentheses.

95% CI= 95% confidence interval.

. The ORs were similar for men and women when analysed separately and also when cases were split according to tumour localisation (colon *vs* rectum) (data not shown).

To further elucidate the relevance of *PTGS2* SNPs, haplotypes for the eight *PTGS2* SNPs were reconstructed using PHASE software (Stephens and Donnelly, 2003). In total, 24 distinct haplo-types were found, six of which with frequencies higher than 1% in the controls. The results are reported in Table 3

**Table 3:** Relative risk of colorectal cancer in relation to *PTGS2* haplotypes

***PTGS2* haplotype^a^**	**Cases/controls^b^**	**OR^c^ (95% CI)**	***P***-**value**
AGCGTTAG	275/268	1	
AGCCTTAG	113/89	1.24 (0.89–1.71)	0.20
GCCGGCAG	89/89	0.97 (0.69–1.37)	0.83
AGCGTCAG	62/66	0.92 (0.62–1.35)	0.65
ACCGGCGA	20/9	2.17 (0.97–4.84)	**0.06**
AGGCTTAG	10/6	1.62 (0.58–4.53)	0.35
GCCGGTAG	2/4	0.49 (0.09–2.68)	0.41
GGCGGCAG	1/2	0.49 (0.04–5.41)	0.56
GCCGTTAG	1/1	0.97 (0.06–15.7)	0.99
			
Rare^d^	12/14	0.84 (0.38–1.84)	0.66

a The order of single-nucleotide polymorphisms (SNPs) in the haplotypes is *PTGS2*.401–*PTGS2.*926–*PTGS2.*1629–*PTGS2.*3050–*PTGS2.*5209–*PTGS2.*8473–*PTGS2.*9850–*PTGS2.*10335.

b Number of chromosomes in each group (double the number of subjects).

c Odds ratio and 95% confidence interval (95% CI).

d Rare haplotypes (frequency ⩽0.5%) that were found only among cases or only among controls.

and showed that the haplotype effects essentially coincide with the effect of *PTGS2*.9850 and *PTGS2*.10335 alleles alone. Indeed, the only haplotype that approaches statistical significance (OR=2.17, 95% CI 0.97–4.84, *P*=0.06) is the one that carries the variant alleles of these two polymorphisms (Table 3).

We explored the relationship between *PTGS2* polymorphisms and exposure to NSAIDs, since these have been shown both to inhibit PTGS2 action *in vitro* and to protect from colorectal cancer. Previous long-term exposure to NSAIDs was clearly protective by itself, especially for NSAIDs different from aspirin (Table 1). Although no statistically significant interaction with *PTGS2*.9850 polymorphism was observed (*P*-value=0.19), the protective effect of NSAIDs observed for the wild-type homozygous genotype (OR for AA=0.55; 95% CI 0.36–0.84) disappeared among those with the variant allele for the polymorphism (OR for AG/GG=1.08, 95% CI 0.17–6.77), since the few exposed with the variant had the same increased risk as those not exposed. The numbers of subjects carrying the variant with NSAID exposure were however very small (six cases and two controls, compared to 18 cases and eight controls not exposed to NSAIDs) and no firm conclusion can be drawn on this point.

## DISCUSSION

In this study, we have found an increased risk for colorectal cancer associated with polymorphisms in *PTGS2*, a gene triggered by the inflammatory response, and governing the synthesis of prostaglandins. Only one polymorphism, at base pair 9850 in GenBank sequence D28235, showed a significant increase in risk. Polymorphism *PTGS2*.10335 also showed a marginally significant association, but *PTGS2*.9850 and *PTGS2*.10335 were in tight LD (*r*^2^=0.79), and we will focus our discussion on 9850, as it is before the poly-A signal, and therefore more likely to have a functional relevance, while 10335 is after. This provides further evidence in the case for the involvement of inflammation as a mechanism of sporadic colorectal cancer.

While the polymorphisms in *PTGS2* have been known to exist for a few years, only one has so far been proven to have any functional effect (Papafili *et al*, 2002). In our study, however, this polymorphism (located at base 926 of GenBank sequence D28235) failed to show an association with colorectal cancer risk. It may be that the acute-phase response that was found associated to this polymorphism is not relevant for long-term mechanisms leading to colorectal carcinogenesis. In addition, a rare variant Val511Ala was reported to be weakly associated with colorectal neoplasia and predicted to modify the conformation of the PTGS2 enzyme (Lin *et al*, 2002). However, this polymorphism is absent in Caucasians and therefore was not included in our study.

The polymorphism at base pair 9850 is located approximately 100 base pairs upstream of the first poly-A signal in *PTGS2*. It can be speculated that this polymorphism has some effect on the addition of the poly-A tail to the mRNA, or causes a later poly-A site to be used, creating a longer, and possibly more stable species of mRNA. An alternative explanation is that it is in LD with other functional polymorphisms nearby. However, we have explored all known frequent polymorphisms of *PTGS2*, up to the SNP located at base 10335, which is downstream of the last polyadenylation site, and the polymorphism at base pair 9850 shows the strongest association. We consider therefore the LD explanation quite unlikely. We have performed haplotype analysis and we believe it reinforces our conclusion on the LD explanation. Indeed, only one haplotype was associated with increased risk, and this is also the only one that carries the two at-risk alleles of SNPs *PTGS2*.9850 and *PTGS2*.10335. Had susceptibility been due to another, yet unidentified polymorphism, we should have observed some increase of risk associated also with other haplotypes, which is not the case. Alternatively, one should postulate the existence of an unknown causal polymorphism in perfect allelic association with *PTGS2*.9850 (i.e. with exactly the same frequency). Such polymorphism could be found by sequencing the entire region between *PTGS2*.9850 and *PTGS2*.10335, and some stretch of sequence upstream and downstream of the two SNPs, in the 20 cases who carry the at-risk haplotype (Table 3). Given that we are dealing with the 3′UTR of the gene, a straightforward interpretation of SNP function cannot be provided by simple analysis of the sequence. Therefore, functional tests should be performed to evaluate *PTGS2.*9850 and any other polymorphism that may be found in the region, in LD with *PTGS2*.9850.

Finally, we have investigated protection from colorectal cancer by NSAIDs and whether it might be modulated by *PTGS2* polymorphisms. Nonsteroidal anti-inflammatory drugs have repeatedly been shown to be protective against colorectal cancer (Gupta and Dubois, 2001). In this study, we find the protective effect stronger for NSAIDs different from aspirin. We attribute this to the fact that aspirin is often consumed at low doses either for sporadic pain relief or for cardiovascular indications. This occasional consumption may be insufficient to confer protection for colorectal cancer. Other NSAIDs, if consumed during a long period as requested in our interview, may have greater preventive potential since the anti-inflammatory power is greater at the doses usually consumed. Although the OR estimates show that the protective effect of NSAIDs disappears for those with the variant allele at *PTGS2*.9850, the interaction is not significant, since the prevalence of NSAIDs exposure is low and combined with the low allele frequency of the variant results in very wide CIs. The hypothesis that protection from colorectal cancer by NSAIDs use might be counteracted by the genetic variant at base 9850 is intriguing. Data from this study can just suggest it but not confirm due to insufficient sample size to study this interaction in detail.

There are several difficulties to face in interpreting the results of case–control studies that explore gene–environment interactions. As in any case–control study, there are limitations involved, like potential selection bias from low participation rates, recall bias, and difficulty in assessing past exposures. We recognise that hospital controls are convenient to get biological material from but are not ideal to recall exposures. We have tried to minimise this potential bias with a careful design, but this may have not been enough. The questionnaire was structured and administered by trained interviewers. From a genetic point of view, we consider the hospitalised controls as being representative of the general Spanish population, as they came from very different hospital departments and included very different diseases. We believe that the impact of hospital controls on the polymorphism status should be minimal, as recently suggested (Garte *et al*, 2001). Moreover, if the proinflammatory allele was related to other diseases that induced hospital admissions, this should have increased its prevalence among controls and biased the ORs towards the null.

In conclusion, we have detected weak associations between several *PTGS2* polymorphisms and risk of colorectal cancer, which reach statistical significance for polymorphism *PTGS2*.9850 (OR=2.49). Possible interactions between this polymorphism and intake of NSAIDs were also detected. The association of *PTGS2* polymorphism with colorectal cancer points towards the implication of inflammation as an important mediator in the carcinogenesis of these tumours. It is worth confirming these findings in other settings and carry out functional studies of the polymorphism.
